# Continuous field flooding versus final one-shot CO_2_ insufflation in minimally invasive mitral valve repair

**DOI:** 10.1186/s13019-022-02020-4

**Published:** 2022-11-01

**Authors:** Giuseppe Nasso, Ignazio Condello, Giuseppe Santarpino, Nicola Di Bari, Marco Moscarelli, Felice Eugenio Agrò, Roberto Lorusso, Giuseppe Speziale

**Affiliations:** 1 Department of Cardiovascular Surgery, Anthea Hospital, GVM Care & Research, Bari, Italy; 2 Department of Cardiac Surgery, Paracelsus Medical University, Nuremberg, Germany; 3 Department of Experimental and Clinical Medicine, “Magna Graecia” University, Catanzaro, Italy; 4 Department of Cardiovascular Surgery, Città di Lecce Hospital, GVM Care & Research, Lecce, Italy; 5 Division of Cardiac Surgery, Department of Emergency and Organ Transplant, Policlinico Hospital, University of Bari, Piazza Giulio Cesare 11, Bari, Italy; 6 Department of Medicine, Unit of Anaesthesia, Intensive Care and Pain Management, Università, Campus Bio-Medico di Roma, Rome, Italy; 7 Cardio-Thoracic Surgery Department, Heart and Vascular Centre, Maastricht University Medical Centre, Maastricht, The Netherlands; 8 Cardiovascular Research Institute Maastricht, Maastricht, The Netherlands

**Keywords:** Carbon dioxide, Minimally invasive cardiac surgery, Mitral valve repair, Transient post-operative cognitive disorder, Cardiopulmonary bypass

## Abstract

**Background:**

Insufflation of carbon dioxide (CO_2_) into the operative field to prevent cerebral or myocardial damage by air embolism is a well known strategy in open-heart surgery. However, here is no general consensus on the best delivery approach.

**Methods:**

From January 2018 to November 2021, we retrospectively collected data of one hundred consecutive patients undergoing minimally invasive mitral valve repair (MIMVR). Of these, fifty patients were insufflated with continuous CO_2_ 1 min before opening the left atrium and ended after its closure, and fifty patients were insufflated with one shot CO_2_ 10 min before the start of left atrium closure. The primary outcome of the study was the incidence of transient post-operative cognitive disorder, in particular agitation and delirium at discontinuation of anesthesia, mechanical ventilation (MV) duration and intensive care unit (ICU) length of stay.

**Results:**

In all patients that received continuous field flooding CO_2_, correction of ventilation for hypercapnia during cardiopulmonary bypass (CPB) was applied with an increase of mean sweep gas air (2.5 L) and monitoring of VCO_2_ changes. One patient vs. 9 patients of control group reported agitation at discontinuation of anesthesia (p = 0.022). MV duration was 14 ± 3 h vs. 27 ± 4 h (p = 0.016) and ICU length of stay was 33 ± 4 h vs. 42 ± 5 h (p = 0.029). A significant difference was found in the median number of total micro-emboli recorded from release of cross-clamp until 20 min after end of CPB (154 in the continuous CO_2_ group vs. 261 in the one-shot CO_2_ control group; p < 0.001). Total micro-emboli from the first 15 min after the release of cross-clamp was 113 in the continuous CO_2_ group vs. 310 in the control group (p < 0.001). In the continuous CO_2_ group, the median number of detectable micro-emboli after CPB fell to zero 9 ± 5 min after CPB vs. 19 ± 3 min in the control group (p = 0.85).

**Conclusion:**

Continuous field flooding insufflation of CO_2_ in MIMVR is associated with a lower incidence of micro-emboli and of agitation at discontinuation of anesthesia, along with improved MV duration and ICU length of stay.

## Introduction

The presence of air micro-emboli in open-heart surgery correlates with the degree of post-operative neuropsychological disorder [1, 2]. Manual de-airing techniques have proved ineffective in eliminating air micro-emboli and even meticulous techniques are associated with the risk of a large number of micro-emboli [3, 4]. The neurological outcome is difficult to evaluate, due to possible bias such as the status and the age of the patient and symptoms (e.g. changes in personality) [5, 6].

However, it is possible that these changes are not connected with the operation itself but rather with the status of the patients, their age, sex, disease severity, or genetic factors [7, 8]. The use of carbon dioxide (CO_2_) in minimally invasive cardiac surgery is due to its high solubility and density in blood, allowing better tolerability of air embolism [9]. The use of endo-cavitary aspirators during mitral valve surgery contributes to capture in the extracorporeal circuit the quantity of CO_2_ continuously insufflated in the surgical field. This aspect is represented in the blood gas analysis and in the frequent correction of hypercapnia through ventilation in the oxygenator [10].

In this context we investigated the effect of CO_2_ on two groups of patients undergoing minimally invasive mitral valve repair (MIMVR) through a right mini-thoracotomy with two different CO_2_ delivery techniques (continuous vs. one end shot) and we compared the peri-operatory micro-embolic activity, the impact of CO_2_ in cardiopulmonary bypass (CPB) management, the incidence of transient post-operative cognitive disorder (TPOCD), mechanical ventilation (MV) duration and intensive care unit (ICU) length of stay.

## Methods

### Patient and data collection

A retrospective, observational study was undertaken of prospectively collected data in one hundred consecutive patients undergoing MIMVR from January 2018 to November 2021 at our Institution Anthea Hospital, GVM Care & Research, Bari, Italy. The median (interquartile range [IQR]) age was 66 (62–76) years, one hundred patients underwent MIMVR through a right thoracotomy approach. Patient characteristics are reported in Table 1. None of the study patients reported the use of psychiatric drugs, alcohol, and carotid artery stenosis prior to the procedure.

**Table 1:** Characteristics of the study population

	All (***n*** = 100)	Continuous CO_2_ (***n*** = 50)	One-shot CO_2_ (***n*** = 50)	***P***-value
Age (years)	66 (62–76)	65 (62–78)	69 (67–79)	< 0.001
Male sex	52.5%	43.9%	38.7%	< 0.001
Body mass index (kg/m^2^)	25.8 (22.2–28.8)	26.8 (23.4–29.0)	24.3 (21.7–28.4)	0.191
Arterial hypertension	32.2%	33.3%	36.1%	0.789
Diabetes mellitus				
Oral antidiabetic drugs	8.9%	7.2%	5.5%	0.006
Insulin	2.3%	2.8%	2.6%	0.530
Hypercholesterolemia	77.4%	38.1%	39.3%	0.899
Renal dysfunction*	1.4%	2.3%	2.1%	0.254
Respiratory or lung disease	2.1%	2.3%	1.9%	0.187
Previous disabling stroke	1.5%	1.5%	1.3%	0.687
History of cancer	1.4%	1.9%	2.5%	0.030
Atrial fibrillation	9.4%	8.3%	11.2%	< 0.001
Peripheral vascular disease	1.8%	1.6%	1.9%	0.556
Coronary artery disease	0.9%	0.8%	1.1%	0.81
LVEF				0.347
>50%	93.0%	92.0%	91.2%	
30–50%	6.3%	7.5%	7.5%	
<30%	0.7%	0.5%	1.3%	
Previous surgery	2.3%	1.7%	1.9%	0.211
EuroSCORE II (%)	1.2 (1.1–2.8)	1.2 (1.1–2.7)	1.2 (1.1–2.8)	0.87

Values are given as median (interquartile range) or percentage

LVEF, left ventricular ejection fraction

*Dialysis or creatinine > 2 mg/dL

Fifty patients were insufflated with continuous CO_2_ 1 min before opening the left atrium and ended after its closure, and fifty patients were insufflated with one shot CO_2_ 10 min before the start of left atrium closure, at a continuous CO_2_ flow rate of 3 L/min via diffuser (Table 1). The main reason for performing two different methods of CO_2_ delivery during MIMVR was due to the different techniques used by cardiac surgeons for minimally invasive cardiac surgery. The aim and the methodology of the study was internal discussed with the ethics committee of the hospital according to the General Data Protection Regulation. Because of the retrospective nature of this study, the local ethics committees waived the need for patient consent. The transesophageal echocardiographic (TEE) protocol for the detection of micro-emboli requires to record intraoperative TEE from cross-clamping to 20 min after end of CPB.

Post-operatively, a blinded assessor determined the maximal number of gas emboli during each consecutive minute in the left atrium, left ventricle, and ascending aorta. The primary outcome of the study was the incidence of TPOCD (in particular agitation and delirium occurring 5 h following weaning from anesthesia), MV duration and ICU length of stay.

During the two procedures, correction for hypercapnia during CPB and monitoring of VCO_2_ changes were recoreded.

### Surgical technique

Our surgical approach for minimally invasive direct view during mitral surgery was described elsewhere [11]. Arterial perfusion was always retrograde and peripheral and aortic cross-clamping was external in all patients. Venous cannulation was peripheral with vacuum support and a double site insertion of the cannulas (jugular and femoral). The valve inspection and procedure were through the left atrium with direct vision and the reconstruction technique was standardized [11].

### CO_2_ insufflation management and CPB de-airing

A small PVC flexible drain tube was used for CO_2_ insufflation as per standardized procedure [12, 13] and flow measurement was performed with a flowmeter for medical CO_2_. The perfusionist regulates the flow according to pCO_2_ and pH. PaO_2_ during CPB was maintained between 150 and 250 mmHg, PaCO_2_ was maintained through the sweep gas (air flow from gas blender) between 40 and 45 mmHg with pH stat management, and mean arterial pressure was maintained between 50 and 70 mmHg [14, 15]. In both groups, the venting flow was maintained 800 ml/min after cross-clamping. Air embolism was managed under TEE guidance; the heart sections were filled, thus obstructing the venous return from CPB and increasing the cavity diameter, and the lungs were manually expanded using an Ambu® resuscitator (Ambu A/S,Ballerup, Denmark) at a rate of 4 inflations per minute. The ventricular and aortic intracavitary aspirators were managed at 750 ml/min and 800 ml/min after cross-clamp removal, and the aortic root vent was removed at the elimination of total gaseous micro-emboli.

### Statistical analysis

Continuous data are expressed as median with IQR and categorical data as percentages. Cumulative survival was evaluated with the Kaplan–Meier method. All reported P-values are two-sided, and P-values of < 0.05 were considered to indicate statistical significance. All statistical analyses were performed with SPSS 22.0 (SPSS, Inc., Chicago, IL, USA).

## Results

CPB duration was 78 ± 13 min and cross-clamp time was 40 ± 9 min (Table 2). The most predominant pathology was degenerative disease, followed by rheumatic mitral valve disease (Table 3). Mitral valve repair was performed in all patients with peripheral cannulation. Repair techniques included annuloplasty, leafleat resection, neochordae implantation and sliding plasty. Table 4 depicts the median number of micro-emboli during the first 15 min after release of the aortic cross-clamp in the three areas of interest taken together. All patients in both groups had micro-emboli after release of the aortic cross-clamp in all three areas of interest. The number of micro-emboli recorded with TEE was higher in the control group (Table 4) and remained constantly higher during all four time periods and in all three studied locations.

**Table 2:** Intraoperative data for surgical techniques and procedures

	Continuous CO_2_ (***n*** = 50)	One-shot CO_2_ (***n*** = 50)	***P***-value
Cardiopulmonary bypass time (min)	79 (65–85)	74 (63–79)	0.98
Cross-clamp time (min)	41 (37–45)	43 (36–47)	0.89

**Table 3:** Mitral valve pathology for minimally invasive mitral valve repair

Mitral valve pathology	Right thoracotomy (***n*** = 100)	Continuous CO_2_ (***n*** = 50)	One-shot CO_2_ (***n*** = 50)
Degenerative	60 (60%)	26 (52%)	34 (68%)
Functional	28 (28%)	17 (34%)	11 (22%)
Rheumatic	12 (12%)	7 (14%)	5 (10%)

**Table 4:** Number of microemboli on transesophageal echocardiographic evaluation of the left atrium and ventricle and the proximal ascending aorta

Time period/area of interest	No. of microemboli		***P***-value
	**Continuous CO**_**2**_ **(n = 50)**	**One-shot CO**_**2**_ **(n = 50)**	
From release of cross-clamp until 20 min after end of CPB			
LA	59 (43–79)	98 (39–129)	< 0.001
LV	49 (45–74)	87 (59–112)	< 0.001
Ao	42 (34–58)	76 (32–88)	< 0.001
LA + LV + Ao	154 (84–195)	261(146–299)	< 0.001
First 15 min after release of cross-clamp			
LA	46 (38–96)	139 (99–159)	< 0.01
LV	65 (58–93)	96 (81–109)	< 0.001
Ao	25 (19–39)	75 (42–89)	< 0.001
LA + LV + Ao	113 (86–157)	310(290–343)	< 0.001
Last 10 min of CPB			
LA	17 (11–43)	27 (9–41)	< 0.01
LV	19 (11–29)	48 (9–59)	< 0.001
Ao	16 (12–37)	36 (25–56)	< 0.01
LA + LV + Ao	52 (42–87)	141(122–156)	< 0.001
First 20 min after end of CPB			
LA	8 (4–21)	48 (19–54)	< 0.01
LV	13 (5–25)	34 (15–41)	0.01
Ao	16 (8–28)	83 (68–98)	< 0.01
LA + LV + Ao	37 (27–58)	165(120–197)	< 0.01

Values are given as median (25th–75th percentile)

Ao, aorta; CPB, cardiopulmonary bypass; LA, left atrium; LV, left ventricle

**Fig. 1:**
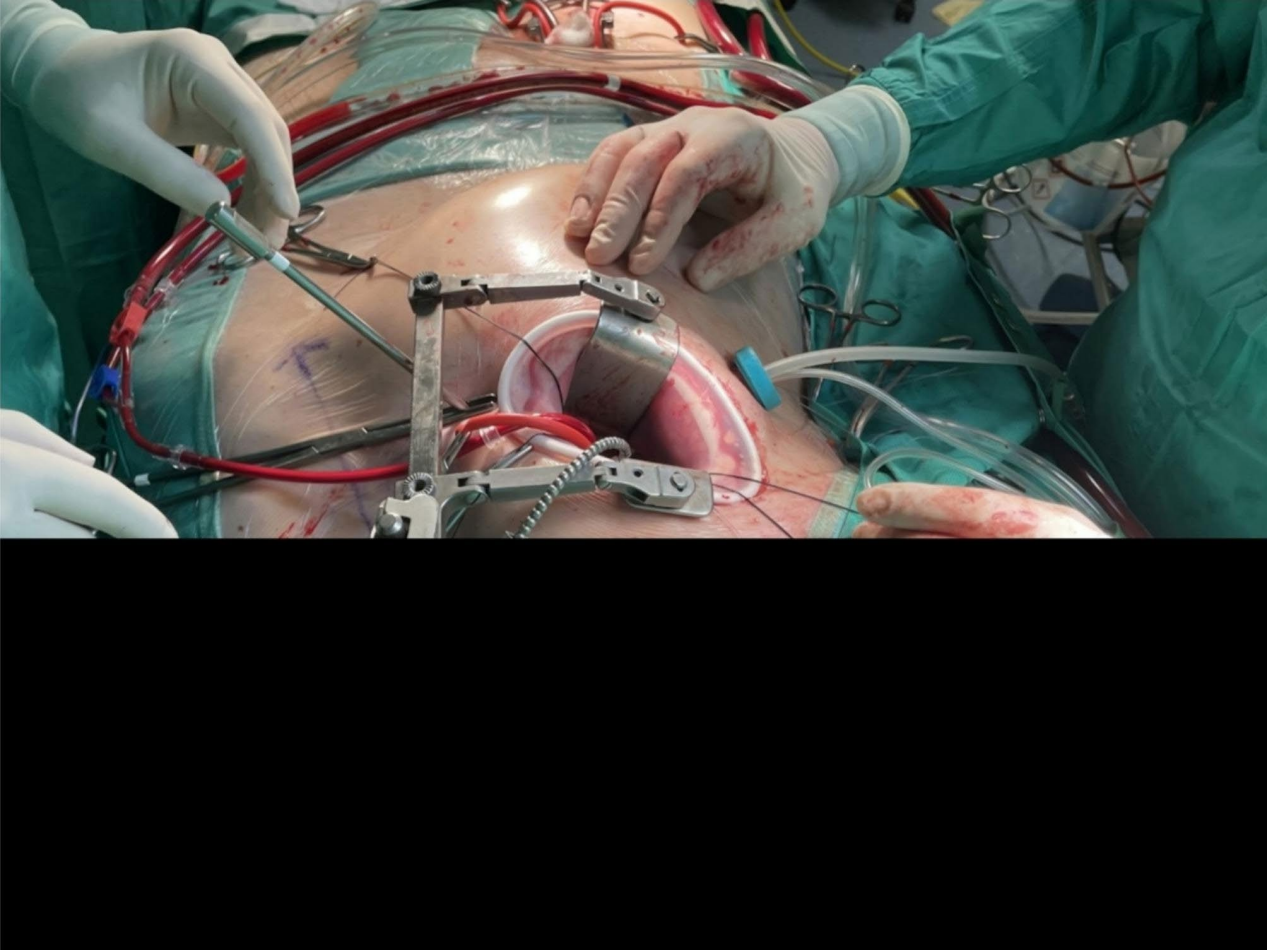
Right thoracotomy for minimally invasive mitral valve repair

In the continuous field flooding CO_2_ group, the median number of detectable micro-emboli after CPB fell to zero 9 ± 5 min after CPB versus 19 ± 3 min in the one-shot CO_2_ control group (p = 0.01). In patients of the continuous field flooding CO_2_ group, correction of ventilation for hypercapnia during CPB was applied, with an increase of mean sweep gas air (2.5 L) and monitoring of VCO_2_ changes. One patient of the continuous CO_2_ group vs. 9 patients of the control group reported agitation at discontinuation of anesthesia (p = 0.022). MV duration was 14 ± 3 h vs. 27 ± 4 h (p = 0.016) and ICU length of stay was 33 ± 4 h vs. 42 ± 5 h (p = 0.029) in the continuous CO_2_ vs. control group, respectively (Table 5). In the whole study population, no transient ischemic attack or stroke was reported at postoperative clinical evaluation (Fig. 1).

**Table 5:** Peri-operative and post-operative outcome

	Continuous CO_2_ (n = 50)	One-shot CO_2_ (n = 50)	***P***-value
Microemboli after CPB fell to zero (min)	9 ± 5	19 ± 3	0.01
Agitation at anesthesia discontinuation (no. of patients)	1	9	0.022
Mechanical ventilation (h)	14 ± 3	27 ± 4	0.016
ICU length of stay (h)	33 ± 4	42 ± 5	0.029

CPB, cardiopulmonary bypass; ICU, intensive care unit

## Discussion

Previous studies [16, 17] demonstrated that the patients without CO_2_ use had persistent air bubbles for many minutes after the end of CPB but these studies were not performed under TEE control, as in our analysis, and no cerebro-vascular outcome was reported [18, 19].

Moreover, subsequent randomized studies showed no difference or were too small to demonstrate a difference in neurocognitive outcome between CO_2_ and no-CO_2_ use [20, 21]. In other words, our study is the first that demonstrate a clinical impact of that strategy.

However, the centrality of TEE use has been previously highlighted for bubble observation [19] but not yet for the clinical outcome effect. Other authors, on the other hand, demonstrated an impact on cardiac function due to less air bubbles in the heart [22]. It should be noted that all these studies tried to compare the use vs. non-use of CO_2_. We are the first that tried to demonstrate a difference in the use of the CO_2_ strategy trying to reduce the possible site effects of CO_2_ (e.g. high pCO_2_) with the support of the perfusionist and a strategy that focuses the use of gas only during the phase of chamber opening. An excess of micro-embolic activity could influence the patient’s awakening by giving drowsiness and transient agitation, this would seem to have an indirect impact on the lack of collaboration by prolonging the time of MV and ICU length of stay.

The main limitation of our study is the quantitative assessment of gaseous micro-embolic activity with a correlation for the primary endpoint of the incidence of TPOCD (in particular agitation and delirium upon discontinuation of anesthesia), MV duration and ICU length of stay, which should be further explored in future studies with instrumental investigations (e.g. magnetic resonance imaging), and be correlated with intraoperative bispectral index, electroencephalogram, and evaluated with cognitive tests in the short, medium and long term in relation to the patient age and gender and the impact of retrograde perfusion and atherosclerotic burden [23].

## Conclusion

Continuous field flooding insufflation of CO_2_ in MIMVR is associated with a lower incidence of micro-emboli, possibly due longer exposure to CO_2_, and a lower incidence of agitation at discontinuation of anesthesia as well as improved MV duration and ICU length of stay.

## Data Availability

The datasets analyzed during the current study are available from the corresponding author on reasonable request.
